# A Multi-Epitope Fusion Protein-Based p-ELISA Method for Diagnosing Bovine and Goat Brucellosis

**DOI:** 10.3389/fvets.2021.708008

**Published:** 2021-09-08

**Authors:** Dehui Yin, Qiongqiong Bai, Xiling Wu, Han Li, Jihong Shao, Mingjun Sun, Jinpeng Zhang

**Affiliations:** ^1^Key Lab of Environment and Health, School of Public Health, Xuzhou Medical University, Xuzhou, China; ^2^Department of Infection Control, The First Hospital of Jilin University, Changchun, China; ^3^Laboratory of Zoonoses, China Animal Health and Epidemiology Center, Qingdao, China

**Keywords:** brucellosis, p-ELISA, serodiagnosis, B cell epitope, bioinformatics technology

## Abstract

In recent years, the incidence of brucellosis has increased annually, causing tremendous economic losses to animal husbandry in a lot of countries. Therefore, developing rapid, sensitive, and specific diagnostic techniques is critical to control the spread of brucellosis. In this study, bioinformatics technology was used to predict the B cell epitopes of the main outer membrane proteins of *Brucella*, and the diagnostic efficacy of each epitope was verified by an indirect enzyme-linked immunosorbent assay (iELISA). Then, a fusion protein containing 22 verified epitopes was prokaryotically expressed and used as an antigen in paper-based ELISA (p-ELISA) for serodiagnosis of brucellosis. The multi-epitope-based p-ELISA was evaluated using a collection of brucellosis-positive and -negative sera collected from bovine and goat, respectively. Receiver operating characteristic (ROC) curve analysis showed that the sensitivity and specificity of detection-ELISA in diagnosing goat brucellosis were 98.85 and 98.51%. The positive and the negative predictive values were 99.29 and 98.15%, respectively. In diagnosing bovine brucellosis, the sensitivity and specificity of this method were 97.85 and 96.61%, with the positive and negative predictive values being identified as 98.28 and 97.33%, respectively. This study demonstrated that the B cell epitopes contained in major antigenic proteins of *Brucella* can be a very useful antigen source in developing a highly sensitive and specific method for serodiagnosis of brucellosis.

## Introduction

Brucellosis, as a re-emerging zoonosis, not only puts human health at risk but also causes tremendous losses in animal husbandry around the world, especially in developing countries (1). Human brucellosis is mainly caused by direct contact with *Brucella*-infected animals or consuming contaminated food (2). In humans, due to the lack of specific clinical manifestations, brucellosis is easily misdiagnosed as other febrile diseases, such as dengue fever, malaria, or viral bleeding diseases (3, 4). In animals, this disease is often neglected as there are no symptoms at the early stage of infection. Therefore, application of diagnostic methods is very important for accurate and early detection of this disease in human and animal populations.

Among the many techniques currently used for diagnosing brucellosis, serological diagnosis methods are the most widely used. It is worth pointing out that accurate serological diagnosis requires highly specific and sensitive antigens (5). However, the current most commonly used antigens for diagnosing brucellosis mainly depend on *Brucella* whole cell and lipopolysaccharide (LPS), which can cross-react with the antibodies aroused by other bacteria, such as *Yersinia enterocolitica* serotype O:9 and *Escherichia coli* O:157. Therefore, it is still meaningful to develop new diagnostic antigens to improve the specificity and sensitivity of serological diagnostic methods for brucellosis (6).

Quite a number of studies showed that the *Brucella* outer membrane proteins (Omps) have good immunogenicity, which can be potentially used as new diagnostic antigens to substitute for LPS (7–9). In this study, B cell epitopes were predicted from these Omps with the help of an online bioinformatics tool, and their capacity in identifying brucellosis-positive sera was further verified. Subsequently, a novel fusion protein containing multiple predicted epitopes was obtained as a candidate antigen for the serodiagnosis of brucellosis. At the same time, using the fusion protein as an antigen, a rapid paper-based enzyme-linked immunosorbent assay (p-ELISA) was constructed and evaluated for its possible use in detecting small ruminant and cattle brucellosis (10).

## Methods

### Serum Samples

A total of 194 goat serum samples (brucellosis-positive sera = 140; brucellosis-negative sera = 54) and 191 bovine sera (brucellosis-positive sera = 116; brucellosis-negative sera = 75) were provided by the China Animal Health and Epidemiology Center (Qingdao, China). All brucellosis-positive sera were confirmed by the Rose Bengal plate agglutination test (RBPT) and tube agglutination test (SAT) according to the national standard for animal brucellosis diagnosis. Negative serum samples were originated from a brucellosis-free area in China. All experiments involving animals or animal samples were fully compliant with ethical approval granted by the Animal Care and Ethics Committee of Xuzhou Medical University (ethical approval no.: 201801W005).

### Prediction and Synthesis of Peptide Epitopes

The amino acid sequences of *Brucella* outer membrane proteins Omp16, Omp25, Omp31, Omp2b, and BP26 were downloaded from NCBI (https://www.ncbi.nlm.nih.gov/protein/). The conserved amino acid sequences were assessed and selected by BLAST. Prediction of B cell epitopes was carried out by online B cell epitope prediction tool BepiPred Linear Epitope Prediction at IEDB (http://tools.iedb.org/bcell/). The predicted B cell epitope peptides were synthesized by Sangon Biotech (Shanghai, China) and coupled to keyhole limpet hemocyanin (KLH) with a productive purity of more than 90%.

### Epitope Verification

Forty-five bovine and goat sera, which were positive for brucellosis, were randomly selected to verify the capability of the predicted peptides in identifying brucellosis through an indirect enzyme-linked immunosorbent assay (iELISA). In addition, KLH was used as negative control and LPS was used as the positive antigen control. For the procedure, in a 96-well microtiter plate (NUNC, Denmark), 100 μL of peptide (30 μg/mL in carbonate buffer solution (CBS), pH 9.6) was added to each well and incubated overnight at 4°C. The wells were blocked with 300 μL/well of 5% skimmed milk powder (Sangon, Shanghai) at 37°C for 2 h, then 100 μL/well of serum was added (1:400 dilution with PBS) and incubated at 37°C for 1 h. HRP-labeled protein G (diluted 1:5,000, PBS) (Thermo, USA) was added and incubated at room temperature for 30 min. After that, an EL-TMB kit was utilized (Sangon) for the coloring step. Optical density was measured at 450 nm (OD450) using an ELISA plate reader (BioTek, USA). During the whole process, plates were washed three times with PBST before each reagent was added.

### Preparation of the Fusion Protein

The effective peptides were connected in random order, and adjacent peptides were linked by the 'GGGS' linker. For the concatenated amino acid sequence, the molecular weight (https://web.expasy.org/compute_pi/), spatial conformation (http://zhanglab.ccmb.med.umich.edu/I-TASSER/), and other parameters were predicted. According to the concatenated sequence, the corresponding codon was designated and optimized for prokaryotic expression. The full length of nucleic acid sequence coding for the multi-epitope fusion protein was synthesized and subcloned into expression vector pET30a (Beijing Protein Innovation, Beijing). The vector was then transferred into competent BL21 cells for IPTG-induced expression. Specifically, competent cells (BL21 cells) (100 μL), stored at−80°C, were slowly thawed on ice, after which the ligation product was added to the cells and mixed well; the cells were then placed on ice for 30 min, heat shocked at 42°C for 90 s, and then incubated in an ice bath for 2 min. Subsequently, 800 μL of non-resistant LB medium was added, incubated at 37°C for 45 min, and centrifuged at 5,000 rpm for 3 min. The majority of the supernatant was discarded, leaving approximately 100–150 μL, which was used to resuspend the cell pellet. The resuspended cells were added to LB plates with the corresponding resistance antibiotic and spread over plates, which were air-dried and cultured upside down and placed in an incubator at 37°C overnight. Then, the transformed BL21 cells were selected and cultured in 1.5 ml of LB liquid medium at 37°C and shaken at 200 rpm. The cells were incubated until OD600 = 0.6, at which time they were induced by IPTG (0.5 mm) and cultured for 2 h at 37°C. 1 ml of induced bacterial solution was centrifuged at 12,000 rpm for 1 min, the supernatant was discarded, and the precipitate was resuspended in 50–100 μL of 10 mM Tris-HCl (pH 8.0) solution (the amount of added buffer was dependent on the amount of bacteria). Loading buffer equal to twice the volume of the resuspended precipitate was added, after which the sample was boiled at 100°C for 5 min and then assessed by SDS-PAGE electrophoresis.

When OD600 of bacterial culture reached 0.6–0.8, IPTG was added to the culture to a final concentration of 0.5 mM and incubated overnight at 16°C. After centrifugation at 6,000 rpm for 5 min, the supernatant was discarded and the precipitation was resuspended in 10 mM of Tris-HCl (pH 8.0) solution. The resuspended bacteria was lysed by ultrasonication (500 W, 60 times, 10 s/each time, 15 s /intervals). The ultrasonic-treated bacterial solution was then centrifuged at 12,000 rpm for 10 min. The supernatant was transferred into another container, and the precipitation was resuspended in 10 mM of Tris-HCl (pH 8.0) solution and assessed by SDS-PAGE electrophoresis.

### Purification of Fusion Protein

The nickel column (Ni Sepharose 6 Fast Flow, GE Healthcare, Uppsala, Sweden) was washed with deionized water at pH 7.0. The nickel column was adjusted to pH 2~3. The column was washed with deionized water at pH 7.0. The nickel column was equilibrated with 10 mM of Tris-HCl (pH 8.0) solution (~100 mL). Then, the nickel column was equilibrated with 10 mM of Tris-HCl (pH 8.0) solution containing 0.5 M of sodium chloride (~50 mL). The diluted sample was loaded. The sample contained sodium chloride at a final concentration of 0.5 M. After loading, the column was washed with 10 mm of Tris-HCl (pH 8.0) solution containing 0.5 M of sodium chloride. The proteins were eluted with 10 mm of Tris-HCl (pH 8.0) (containing 0.5 M of sodium chloride) solution containing 15 mm of imidazole, 60 mM of imidazole, and 300 mm of imidazole, and the protein peaks were collected separately. SDS-PAGE electrophoresis was used to assess the protein purity.

### Antigenicity Assessment of the Fusion Protein

iELISA was used to assess the capability of purified protein in identifying brucellosis-positive sera. In a 96-well ELISA plate (NUNC, Denmark), 100 μL of fusion protein (2.5 μg/mL in CBS) and 100 μL of LPS (1 μg/mL in CBS) as the positive antigen control were added to the wells, respectively, and incubated overnight at 4°C. In the blocking step, 300 μL of 5% skimmed milk (PBS) was added per well and incubated at 37°C for 2 h. Then, 100 μL of serum (1:400 dilution in PBS) was added and incubated at 37°C for 1 h. After that, 100 μL of HRP-labeled protein G (diluted 1:8,000 in PBS) was added and incubated at room temperature for 30 min. When the coloring step was finished with the EL-TMB kit, the absorbance of the wells was measured at OD450. After each step, the plates were washed three times with PBST.

In addition, rabbit sera confirmed to be infected with *Yersinia enterocolitica* O:9, *Escherichia coli* O157:H7, *Salmonella, Vibrio cholerae, Vibrio parahaemolyticus*, and *Listeria monocytogenes* were used to assess the specificity of the fusion protein antigen. All these rabbit sera were purchased from Tianjin Biochip Corporation (Tianjin, China). The verification method was the same as iELISA described above except a 1:10,000 dilution of HRP-labeled goat anti-rabbit secondary antibody (Bioworld, USA) was used in this assay.

### Establishment of the p-ELISA Method

A round sheet with a diameter of 10 mm was punched from Whatman No. 1 filter paper, and a small hole (6 mm diameter) was punched out of A4 plastic packaging paper. The 10 mm filter paper was placed in the center of the 6 mm hole in the plastic packaging paper, and a laminating machine was used to join the filter sheet and packaging paper together, and then the combined papers were fixed and cut into small strips with three holes in each strip. The following steps were conducted according to the literature (11): 5 μl of chitosan in deionized water (0.25 mg/ml) was added to the round holes with Whatman No. 1 filter paper and dried at room temperature; then, 5 μl of 2.5% glutaraldehyde solution in PBS was added and incubated at room temperature for 2 h. After washing three times with 20 μl of deionized water, 5 μl of fusion protein solution (2.5 μg/ml in PBS) was added to each well and incubated at room temperature for 30 min. After another three washes with 20 μl of deionized water, 20 μl of 5% skimmed milk powder was added and incubated at room temperature for 15 min. Subsequently, 5 μl of serum (1:400 dilution) and 5 μl of HRP-labeled protein G (1:8,000 dilution) were added in order and washed three times with PBST at intervals. Finally, 5 μl of TMB substrate solution was added and incubated for 10 min, then a HP Laser Jet Pro MFP M227 was used to scan the samples to obtain images. ImageJ software was used to perform gray intensity analysis for quantitation. The cattle and goat serum samples were assessed according to the established *p*-ELISA method, and ROC curves were used to analyze the diagnostic effect of the established method.

### Statistical Analysis

Dot plot and receiver operating characteristic curve (ROC) analyses were performed using GraphPad Prism version 6.05 for Windows. The significance of gray intensity differences was determined by Student's *t*-test (unpaired *t*-test). Differences were considered statistically significant when *P* < 0.05.

## Results

### B Cell Epitope Peptide Prediction and Antigenicity Verification

**Figure 1:**
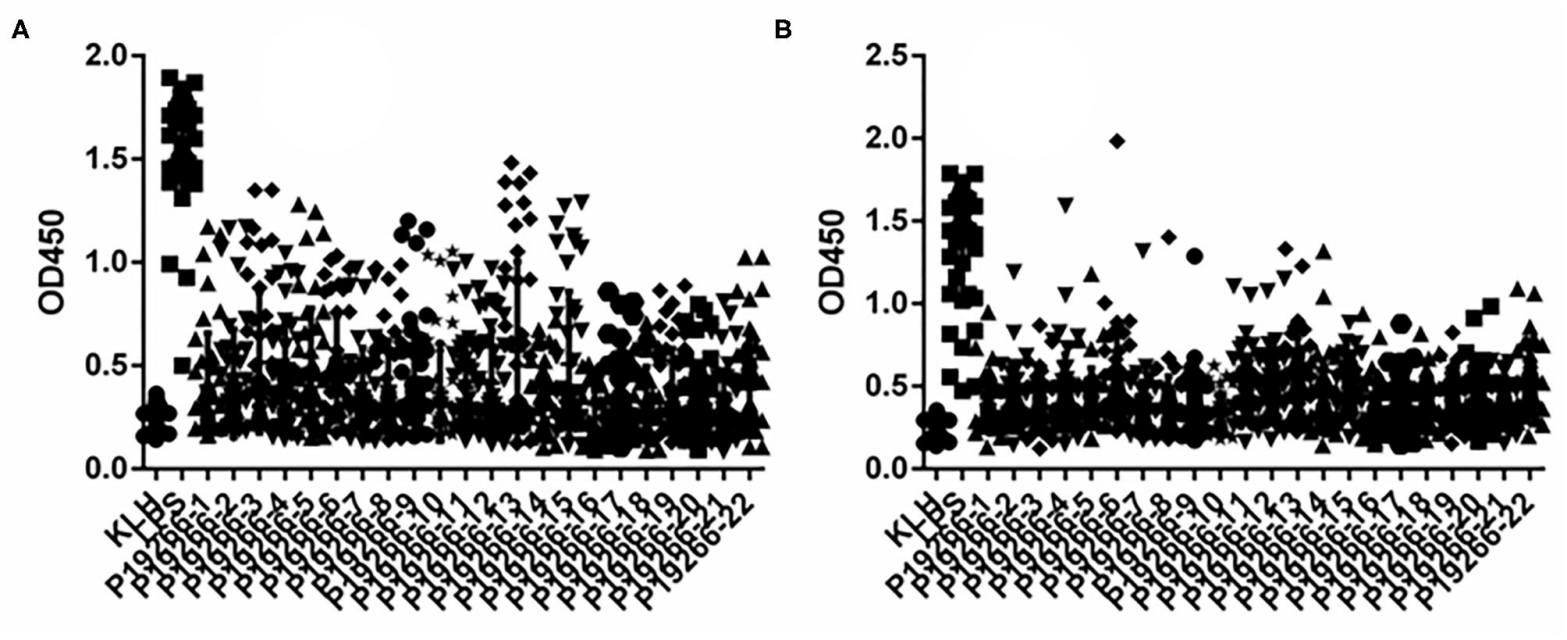
The results of iELISA of each peptide identification-positive brucellosis serum. **(A)** Sheep brucellosis serum. **(B)** Bovine brucellosis serum.

From 5 Omps, a high number of epitopes were predicted by BepiPred software with the length of peptides ranging from 1 to 28. Empirically, only peptides longer than six amino acids were chosen as candidate epitopes. Thus, a total of 22 B cell epitopes were selected and synthesized for subsequent verification analysis, including six peptides from BP26, two from Omp16, five from Omp25 and Omp31 respectively, and four from Omp2b (Supplementary Table 1). Indirect ELISA results showed that all 22 peptides demonstrated some extent of capability in identifying animal-sourced brucellosis-positive sera (Figure 1).

### Preparation of the Multi-Epitope Fusion Protein

**Figure 2:**
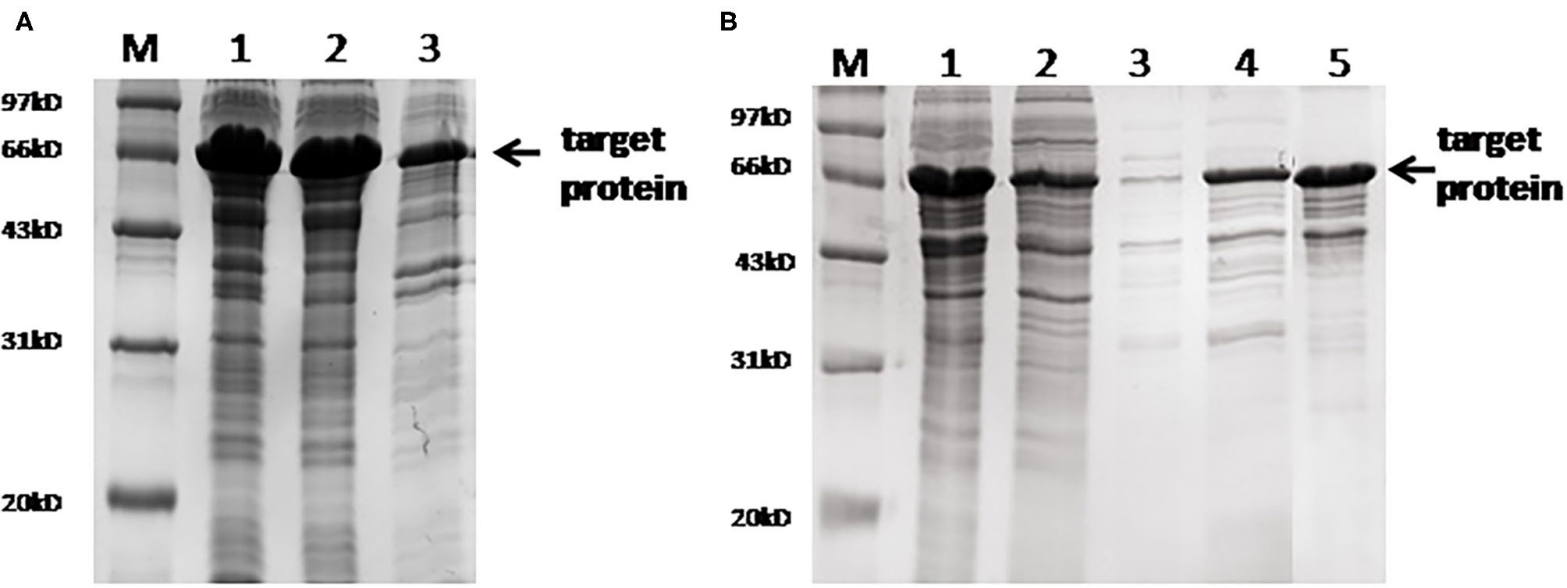
SDS-PAGE analysis of the fusion protein. **(A)** Protein expression results. M, marker; lane 1, whole bacteria after ultrasound; lane 2, supernatant after ultrasound; lane 3, precipitation after ultrasound. **(B)** SDS-PAGE after protein purification. M, marker; lane 1, the original protein before purification; lane 2, flow-through solution; lane 3, 15 mm of imidazole elution fraction; lane 4, 60 mm of imidazole elution fraction; lane 5, 300 mm of imidazole elution fraction.

The full sequence of the fusion protein containing 22 epitopes and 'GGGS' linker is listed in Supplementary Figure 1. This fusion protein was successfully expressed in the soluble form in the prokaryotic system. SDS-PAGE electrophoresis showed that the molecular weight of the purified fusion protein was ~66 kd. Mass spectrometry analysis confirmed that the sequence of the purified protein was identical to the designed target. Gray intensity analysis showed that the purity of the purified protein was ~90% (Figure 2).

### Antigenicity Assessment of the Fusion Protein

**Figure 3:**
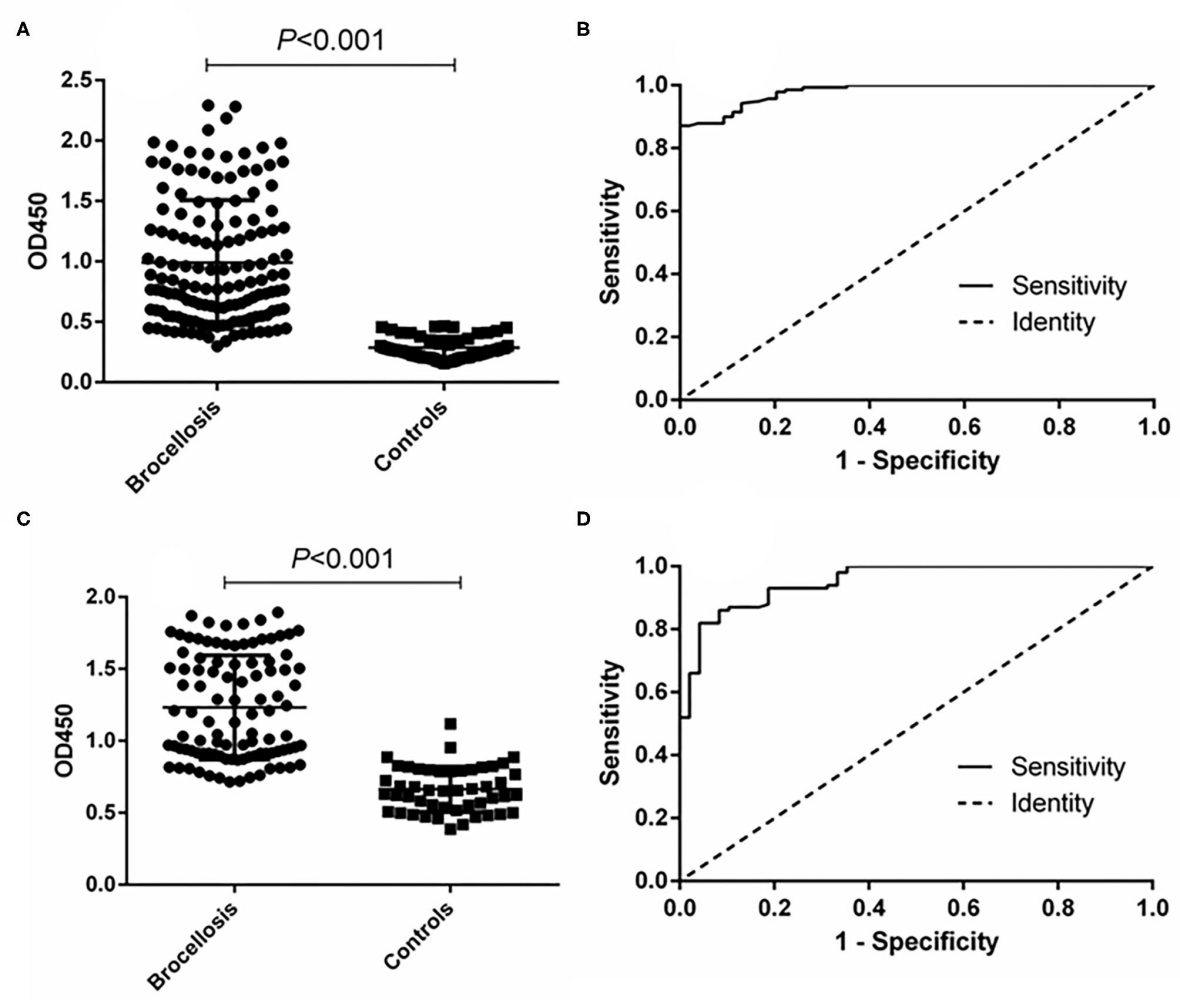
ELISA analysis of goat serum samples. **(A)** Dot plot of the fusion protein ELISA assay. **(B)** ROC analysis of fusion protein iELISA assay results. **(C)** Dot plot of the LPS antigen ELISA assay. **(D)** ROC analysis of LPS antigen ELISA assay results.

The ability of the fusion protein in diagnosing goat brucellosis was evaluated using 140 sera with a known *Brucella* infection background and 54 sera negative control sera by the method of iELISA. According to ROC curve analysis, the area under the ROC curve was 0.9799 (95% CI, 0.9654 to 0.9944), and the cutoff value calculated by the Youden index was 0.4675. In this case, the diagnostic sensitivity was 87.14% (95% CI, 0.8044 to 0.9220), and the specificity was 100.0% (95% CI, 0.9340 to 1.000). The positive predictive value was 100.0%, and the negative predictive value was 75.00% (Figure 3 and Table 1). In LPS control experiments, the area under the ROC curve was 0.9514 (95% CI, 0.9191 to 0.9836), and the cutoff value was 0.8890. At this cutoff value, the diagnostic sensitivity was 82.00% (95% CI, 0.7305 to 0.8897) and the specificity was 95.83% (95% CI, 0.8575 to 0.9949). The positive predictive value was 98.39%, and the negative predictive value was 74.29% (Figure 3, Table 1).

**Table 1:** Positive and negative predictive values of the test calculated for different cutoff values.

**Cutoff value**	**Positive**		**Negative**		**PPV (%)**	**NPV (%)**
	**TP**	**FN**	**TN**	**FP**		
≥0.4675 (fusion protein)^a^	122	18	54	0	100.0	75.00
≥0.8890 (LPS)a	122	18	52	2	98.39	74.29
≥0.4530 (fusion protein)^b^	103	13	70	5	95.37	84.34
≥0.8105 (LPS)^b^	107	9	68	7	93.86	88.31
≥34.12 (*p*-ELISA)^a^	139	1	53	1	99.29	98.15
≥30.21 (*p*-ELISA)^b^	114	2	73	2	98.28	97.33

*a, goat sera; b, cattle sera. TP, true positives; TN, true negatives; FP, false positives; FN, false negatives; PPV, positive predictive value (TP/TP+FP) ×100; NPV, negative predictive value (TN/TN+FN) ×100*.

**Figure 4:**
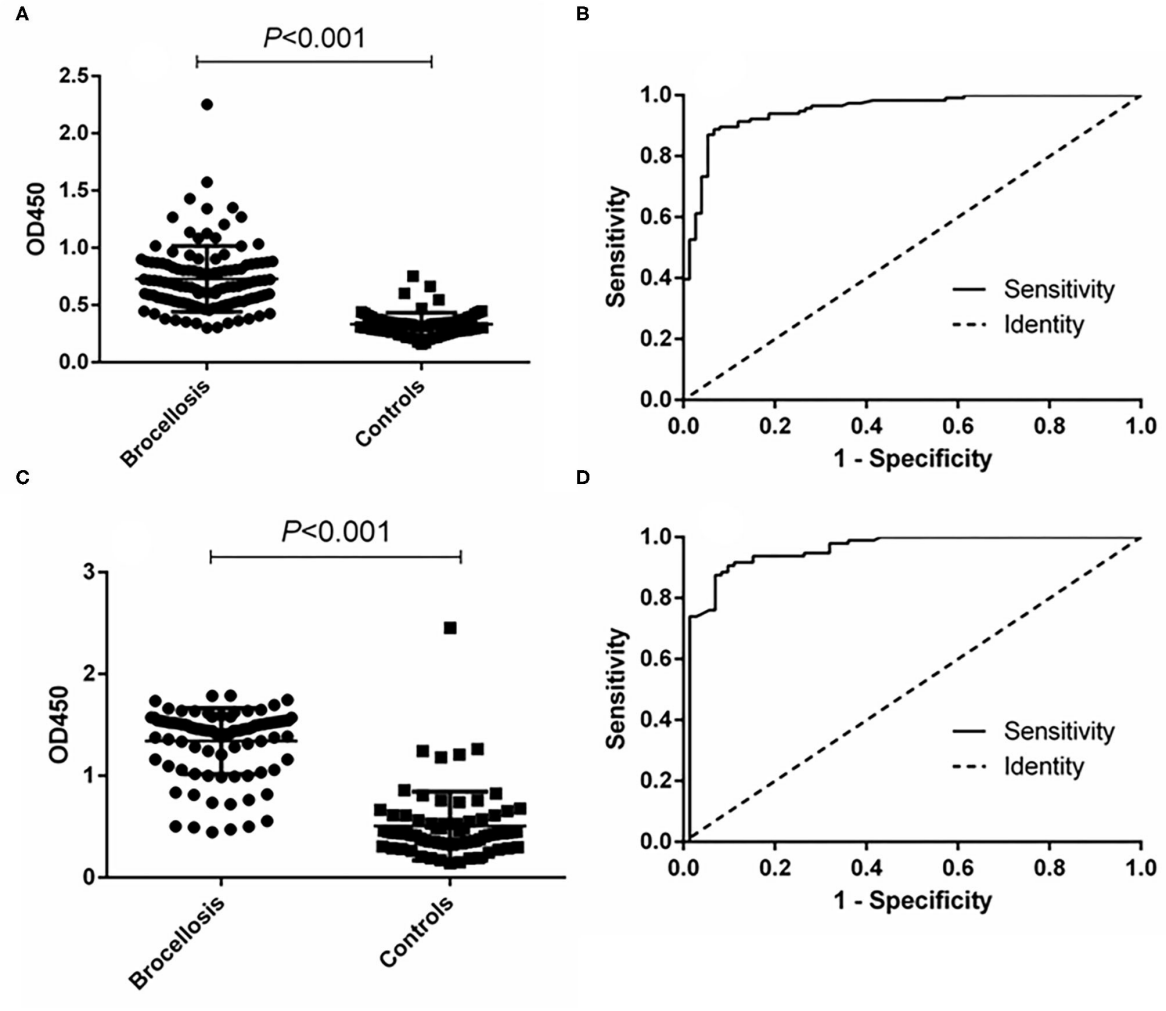
ELISA analysis of cattle serum samples. **(A)** Dot plot of the fusion protein ELISA assay. **(B)** ROC analysis of fusion protein iELISA assay results. **(C)** Dot plot of the LPS antigen ELISA assay. **(D)** ROC analysis of LPS antigen ELISA assay results.

In the cattle brucellosis experiment using 191 cattle sera with a known infection background, the area under the ROC curve was 0.9518 (95% CI, 0.9224 to 0.9812), and the cutoff value calculated by the Youden index was 0.4530. In this case, the diagnostic sensitivity was 88.79% (95% CI, 0.8160 to 0.9390), and the specificity was 93.33% (95% CI, 0.8512 to 0.9780) (Figure 4 and Table 1). The positive predictive value was 95.37%, and the negative predictive value was 84.34%. When using LPS as the antigen, the area under the ROC curve was 0.9528 (95% CI, 0.9187 to 0.9868) and the cutoff value was 0.8105. In this case, the diagnostic sensitivity was 90.63% (95% CI, 0.8295 to 0.9562) and the specificity was 90.28% (95% CI, 0.8099 to 0.9600). The positive predictive value was 93.86%, and the negative predictive value was 88.31% (Figure 4, Table 1).

### Determining the Cross-Reactivity With the Fusion Protein

To verify whether the fusion protein as a diagnostic antigen showed cross-reactivity with other bacteria, we selected six zoonotic pathogens for a cross-reactivity test. The results showed that the fusion protein did not cross-react with other bacteria according to an S/N (OD450, sample/negative) > 2.1, which indicated that the fusion protein antigen had better specificity (Table 2).

**Table 2:** Specific cross-reactivity test results of the indirect ELISA diagnostic method for the fusion protein.

**Rabbit sample**	**OD450**	**S/N**
*Vibrio parahaemolyticus*	0.1230	1.64
*Escherichia coli* O157:H7	0.0457	0.61
*Salmonella*	0.1267	1.69
*Vibrio cholerae*	0.0598	0.80
*Yersinia enterocolitica*O9	0.0443	0.59
*Listeria monocytogenes*	0.0758	1.01
Negative	0.0751	-

### Evaluation of the Diagnostic Ability of the p-ELISA

**Figure 5:**
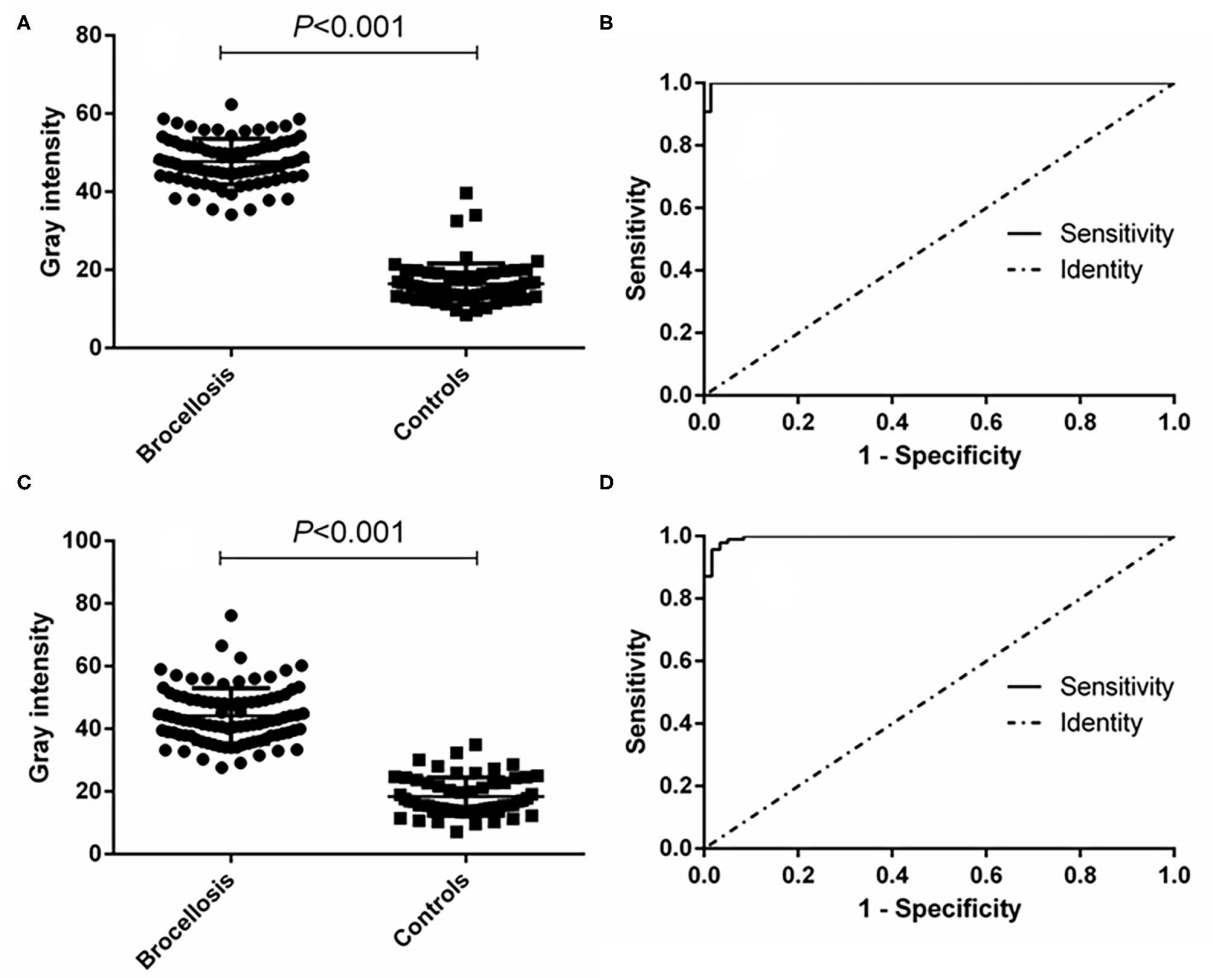
p-ELISA analysis of goat and cattle samples. **(A)** Dot plot of the goat samples. **(B)** ROC analysis of goat samples. **(C)** Dot plot of the cattle samples. **(D)** ROC analysis of cattle samples.

The effectiveness of the established p-ELISA method in detecting animal brucellosis was also evaluated. When it was used for diagnosing goat sera, the area under the ROC curve was 0.9986 (95% CI, 0.9957 to 1.002). The cutoff value was 34.12, at which the diagnostic sensitivity was 98.85% (95% CI, 0.9376 to 0.9997) and the specificity was 98.51% (95% CI, 0.9196 to 0.9996). The positive predictive value was 99.29% and the negative predictive value was 98.15% (Table 1). When it was used for diagnosing cattle brucellosis, the area under the ROC curve was 0.9964 (95% CI, 0.9910 to 1.002), and the cutoff value calculated by the Youden index was 30.21. In this case, the diagnostic sensitivity was 97.85% (95% CI, 0.9245 to 1.002) and the specificity was 96.61% (95% CI, 0.8829 to 0.9959). The positive predictive value was 98.28%, and the negative predictive value was 97.33% (Figure 5, Table 1).

## Discussion

Brucellosis is a serious zoonotic disease. Bovine and small ruminants are the most susceptible animals (12). Currently, culling infected animals is an effective strategy to prevent this disease from spreading (13). Thus, accurate diagnosis would be very important to pick out truly *Brucella*-infected animals and reduce unnecessary economic losses. Particularly in China, where a large number of bovine and goat are raised, fast and efficient methods for brucellosis are of great significance (14). Serological diagnostic techniques are mainly used for brucellosis detection, including the agglutination test, complement fixation test (CFT), ELISA, immunochromatographic diagnostic test (ICDT), and fluorescence polarization assay (FPA) (15, 16). But, these methods normally use *Brucella*-derived LPS as the diagnostic antigen, and a false positive result can be easily produced as *Brucella* LPS shares a common antigenic epitope with other pathogens such as *Escherichia coli* O157:H7 and *Yersinia enterocolitica* O9 (17). In addition, LPS antigen is only obtained by culturing live *Brucella*, which greatly reduces its availability. Therefore, seeking more specific and easily accessible antigens is still meaningful for brucellosis diagnostics research.

ELISA is currently the most widely studied serological diagnosis method, even as diagnostic confirmation in brucellosis (16). The main problem with using ELISAs for the diagnosis of brucellosis is the choice of antigen, but to date, ELISA-based diagnoses lack a single standard antigen (18). Currently, the most commonly used diagnostic antigens used in ELISA are whole bacteria or extracts. These diagnostic antigens are prone to cross-reactivity with other bacteria, have poor specificity, and have considerable defects. Therefore, the development of new diagnostic antigens is key to improving the diagnostic effect of ELISAs.

The *Brucella* Omps are a group of proteins with various molecular weights (19). Some Omps have been identified to be able to arouse strong immune responses in infected animals, including Omp16, Omp25, BP26, Omp2b, and Omp31. In this study, Omp16, Omp25, BP26, Omp2b, and Omp31 were selected for prediction of B cell epitopes and construction of a new diagnostic antigen. Omp16 is a lipoprotein that can elicit immune response and can be potentially used in diagnostics and vaccine development (8, 20). Omp25 plays an important role in Brucella pathogenesis during infection, and exhibits strong immunogenicity (21). A subunit vaccine comprising BP26 triggers a mixed Th1/Th2 immune response in a mice model (7), and it has been also used in diagnosis of brucellosis (22). Animal experiments indicated that Omp31 can not only elicit a strong humoral immune response in mice, but also protects mice against *Brucella* infection (23, 24). Omp2b is another important candidate for brucellosis diagnostics and vaccine research (25, 26). The data in this paper proved that the shorter linear peptides contained in these Omps are also effective in detecting brucellosis-positive sera. In addition, better effectivity can be achieved by using multiple epitopes, as data showed that a single epitope only identified partial serum samples while a multi-epitope fusion protein detected almost all the positive sera. More importantly, the specificity of a multi-epitope protein antigen was higher than that of LPS, implying that the method using the multi-epitope antigen can be used as a confirmatory diagnosis method for brucellosis. It is worth pointing out that bioinformatics tools applied in this study are very helpful to predict effective antigens (27, 28), in the future, more novel antigens can be prepared using this strategy.

The *p*-ELISA method using paper as the solid-phase carrier is a new technology developed based on the traditional ELISA method (11, 29). Compared with the traditional ELISA method, *p*-ELISA is faster, less reagent is required, and no special instruments are needed (30). Currently, the most commonly used paper-processing method for *p*-ELISA involves preparing hydrophilic and hydrophobic areas through wax-printing technology. This method requires expensive printers, which limits the application of this method. We used plastic-encapsulated paper to prepare a hydrophobic area, punched small holes in it, and filled the small holes with hydrophilic paper sheets to make a sandwich structure. This modification greatly reduced the production cost. Combing the multi-epitope-based fusion protein as the antigen, our *p*-ELISA demonstrated improved sensitivity and specificity in diagnosing cattle and small ruminant brucellosis. This newly developed p-ELISA method is more suitable for rural areas where animal brucellosis is highly epidemic and experiment equipment is unavailable.

In China, animal immunization by *Brucella* vaccine has been carried out in some provinces. A serological method for distinguishing infected from vaccinated animals (DIVA) is urgently needed. As the sera used in this study were collected from wild-type *Brucella*-infected animals, the DIVA ability of the multi-epitope-based p-ELISA is not known. Further research will be carried out to determine whether this method can be applied to test other animal or human brucellosis or used for DIVA purposes.

## Data Availability Statement

The original contributions presented in the study are included in the article/Supplementary Material, further inquiries can be directed to the corresponding authors.

## Ethics Statement

The animal study was reviewed and approved by Animal Care and Ethics Committee of Xuzhou Medical University.

## Author Contributions

DY and QB analyzed the data and drafted the manuscript. QB, XW, and HL performed the assays. JS reviewed and made improvements in the manuscript. DY, MS, and JZ conceived and designed the study. All authors read and approved the final version of the paper.

## Funding

This work was supported by the National Natural Science Foundation of China (grant number 81802101). The funders had no role in the study design, data collection and analysis, decision to publish, or preparation of the manuscript.

## Conflict of Interest

The authors declare that the research was conducted in the absence of any commercial or financial relationships that could be construed as a potential conflict of interest.

## Publisher's Note

All claims expressed in this article are solely those of the authors and do not necessarily represent those of their affiliated organizations, or those of the publisher, the editors and the reviewers. Any product that may be evaluated in this article, or claim that may be made by its manufacturer, is not guaranteed or endorsed by the publisher.
