# Supplementary material for: A Multi-Epitope Fusion Protein-Based p-ELISA Method for Diagnosing Bovine and Goat Brucellosis
Source: Front Vet Sci. 2021 Sep 8;8:708008. doi: 10.3389/fvets.2021.708008 (PMC8455990; doi:10.3389/fvets.2021.708008)
Supplement: Supplementary file 1 [file Data_Sheet_1.doc]

**A Multi-Epitope Fusion Protein-Based p-ELISA Method for Diagnosing Bovine and Goat Brucellosis**

Dehui Yin^1†^, Qiongqiong Bai^1†^, Xiling Wu^1^, Han Li^2^, Jihong Shao^1^, Mingjun Sun^3*^, Jingpeng Zhang^1*^

^1^ Key Lab of Environment and Health, School of Public Health, Xuzhou Medical University, Xuzhou, 221004, China

^2^Department of Infection Control, the First Hospital of Jilin University, Changchun, 130021, China

^3^ Laboratory of Zoonoses, China Animal Health And Epidemiology Center, Qingdao, 266032, China

^†^ These authors contributed equally to this work

*Correspondence:

Laboratory of Zoonoses, China Animal Health And Epidemiology Center, No.369 Nanjing Road, Qingdao, 266032, China. E-mail: sunmingjun@cahec.cn & School of Public Health, Xuzhou Medical University, No. 129 Tongshan Road, Xuzhou, 221004, China. E-mail: xiaopangpeng@126.com

**Table S1.** Detailed information of 22 predicted B cell epitopes.

| **Protein** | **Epitope (amino acid sequence)** | **Start-end position** | **Peptide ID** |
| --- | --- | --- | --- |
| BP26 | AFAQENQMTTQPARIAV | 26-42 | P19266-1 |
|  | KAGIEDRDLQTGGIN | 88-100 | P19266-2 |
|  | QPIYVYPDDKNNLKEPTITGY | 104-124 | P19266-3 |
|  | GVNQGGDLNLVNDNPSAVIN | 151-170 | P19266-4 |
|  | LSRPPMPMP | 204-212 | P19266-5 |
|  | AAAPDNSVPIAAGENSYNVSVNVVFE | 223-248 | P19266-6 |
| Omp2b | SGAQAADAIVAPEPEAVEY | 31-49 | P19266-7 |
|  | DVKGGDDVYSGTDRNGWDK | 79-97 | P19266-8 |
|  | NNSGVDGKYGNETSSGTV | 129-146 | P19266-9 |
|  | TVTPEVSYTKFGGEWKNTVAEDNAWGGI | 341-368 | P19266-10 |
| Omp16 | AAAPGSSQDFTV | 44-55 | P19266-11 |
|  | SRGVPTNRMRTISYGNERPVAVCD | 125-148 | P19266-12 |
| Omp25 | GRAKLENRTNGGTS | 56-69 | P19266-13 |
|  | GNPVQTTGETQ | 115-125 | P19266-14 |
|  | GGIKNSLRIGGEESSKSKTQT | 154-174 | P19266-15 |
|  | GWTVGAGIEYAA | 175-186 | P19266-16 |
|  | TDYGKKNFGLNDLDTRGSFKTNDIR | 199-223 | P19266-17 |
| Omp31 | VSEPSAPTAAPVDTFSWTGGYIGINA | 24-49 | P19266-18 |
|  | GKFKHPFSSFDKEDNEQVSGSL | 53-75 | P19266-19 |
|  | TGSISAGASGLEGKAE | 112-127 | P19266-20 |
|  | GDDASALHTWSDKTKAGWTLGAGAEYA | 168-194 | P19266-21 |
|  | DLGKRNLVD | 109-217 | P19266-22 |

**
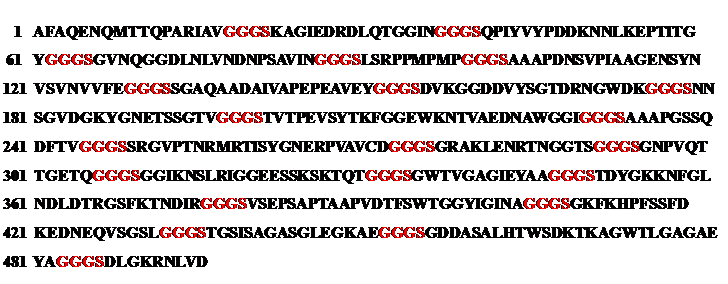
**

Fig. S1 Comprehensive sequence of amino acids of the fusion protein; the linkers are in red font.


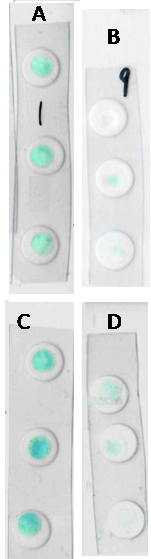


**Fig. S2 Results of p-ELISA.** (A) Positive of goat sera. (B) Negative of goat sera. (C) Positive of bovine sera. (D) Negative of bovine sera.
